# Lactoferrin impairs pathogen virulence through its proteolytic activity

**DOI:** 10.3389/fvets.2024.1428156

**Published:** 2024-08-08

**Authors:** Ruben Ongena, Matthias Dierick, Daisy Vanrompay, Eric Cox, Bert Devriendt

**Affiliations:** ^1^Laboratory of Immunology, Department of Translational Physiology, Infectiology and Public Health, Faculty of Veterinary Medicine, Ghent University, Merelbeke, Belgium; ^2^Laboratory for Immunology and Animal Biotechnology, Department of Animal Sciences and Aquatic Ecology, Faculty of Bioscience Engineering, Ghent University, Ghent, Belgium

**Keywords:** lactoferrin, antimicrobial, proteolytic activity, bacterial virulence factors, pathogenicity

## Abstract

Antibiotics, often hailed as ‘miracle drugs’ in the 20th century, have revolutionised medicine by saving millions of lives in human and veterinary medicine, effectively combatting bacterial infections. However, the escalating global challenge of antimicrobial resistance and the appearance and spread of multidrug-resistant pathogens necessitates research into alternatives. One such alternative could be lactoferrin. Lactoferrin, an iron-binding multifunctional protein, is abundantly present in mammalian secretions and exhibits antimicrobial and immunomodulatory activities. An often overlooked aspect of lactoferrin is its proteolytic activity, which could contribute to its antibacterial activity. The proteolytic activity of lactoferrin has been linked to the degradation of virulence factors from several bacterial pathogens, impeding their colonisation and potentially limiting their pathogenicity. Despite numerous studies, the exact proteolytically active site of lactoferrin, the specific bacterial virulence factors it degrades and the underlying mechanism remain incompletely understood. This review gives an overview of the current knowledge concerning the proteolytic activity of lactoferrins and summarises the bacterial virulence factors degraded by lactoferrins. We further detail how a deeper understanding of the proteolytic activity of lactoferrin might position it as a viable alternative for antibiotics, being crucial to halt the spread of multi-drug resistant bacteria.

## 1 Introduction

Lactoferrin (LF), a multifunctional iron-binding glycoprotein belonging to the transferrin family, is widely known for its antimicrobial activity against a broad range of pathogens (1). LF was first discovered in bovine milk in 1939 and was found in human milk in 1960 (2–4). This mammalian glycoprotein is most abundant in milk and colostrum but is also present in other body fluids such as tears, saliva, vaginal mucus, seminal plasma and bile (5). LF can also be found in the secondary granules of neutrophils, where it is released upon infection (6). The concentration of LF in milk and colostrum varies by species, with humans and bovines having the highest concentrations at around 8 and 1.5 g/L for colostrum, respectively, and 1.5–4 g/L and around 0.2 g/L for milk. LF is also found in the exocrine secretions of other mammals, such as pigs, goats, buffalos, horses, camels, and mice (5, 7, 8). A sequence identity of ±70% is reported across these different species, corresponding with a highly conserved 3D structure, making it interesting to study and compare the functions of LF in different organisms (9).

**Figure 1:**
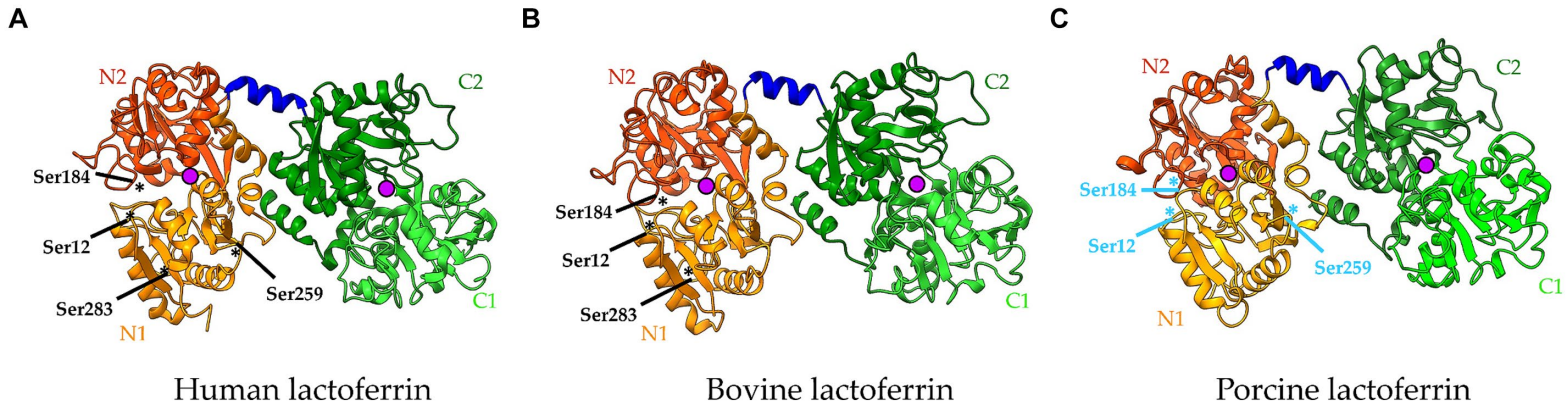
Cartoon representation of the 3D structure of **(A)** human lactoferrin (PDB code = 1B0L), **(B)** bovine lactoferrin (PDB code = 1BLF), and **(C)** porcine lactoferrin (Uniprot ID = P14632). The lactoferrin molecule consists of two lobes, the N-lobe (highlighted in orange) and the C-lobe (highlighted in green), connected by an α-helix structure (highlighted in dark blue). Each lobe can be further subdivided into two domains: N1, N2, C1, C2. Each lobe contains an iron (Fe^3+^)-binding site (purple spheres). Serine residues that were previously identified as possible candidates responsible for the protease activity of lactoferrin are indicated in black, while serine residues that are putatively involved are indicated in light blue. The location of these residues is indicated with an asterisk. Figures were created using UCSF Chimera X v. 1.6.1 software.

LF is an 80 kDa polypeptide comprised of two highly homologous globular lobes, a C-and an N-lobe, connected by a hinge with an α-helical structure (Figure 1). Both the C-and N-lobe can be further separated into two domains, resulting in a C1, C2, N1, and N2 domain, with a deep cleft in each lobe separating these domains (10). This interdomain cleft contains an iron-binding site, where one Fe^3+^ atom can bind. LF can thus influence iron homeostasis. Since the binding of iron is reversible, LF exists in both an iron-free (apo-lactoferrin) and iron-saturated (holo-lactoferrin) form (11, 12).

As a host defence protein, LF protects the host against infections with bacteria, viruses, fungi, and parasites (12). The antibacterial activity of LF is one of its most well-known and well-documented characteristics. This activity can be divided into a bacteriostatic and a bactericidal mode of action against both Gram− and Gram+ bacteria. LF functions as a bacteriostatic agent by sequestering iron in the bacterial environment, thereby depriving bacteria of this essential nutrient, limiting their growth (11). The bactericidal mode of action of LF operates by binding cell wall components such as lipopolysaccharide and lipoteichoic acid, enabling a direct interaction of LF with both Gram− and Gram+ bacteria, hereby destabilising the bacterial membrane, resulting in bacterial cell death (13, 14). LF’s antibacterial activity has been demonstrated against several bacterial organisms such as *Escherichia coli*, *Staphylococcus aureus*, *Bacillus subtilis*, *Haemophilus influenzae*, *Vibrio cholerae*, *Chlamydia psittaci,* and *Listeria monocytogenes* (15–20).

Not only LF, but also LF-derived peptides such as lactoferricins possess an antibacterial activity, sometimes even exceeding that of the native protein (21). Lactoferricins (Lfcins) are antimicrobial peptides derived from the N-terminus of LF and are generated by acidic proteolysis, such as via the gastric enzyme pepsin. Consequently, these can be found in the stomach and throughout the gastrointestinal tract (22). Lfcins are already identified both in bovines (amino acid 17–41) and in humans (amino acid 1–47) and these were shown to not only display a greater antibacterial activity than LF but can also exert antifungal and antiparasitic activities (21, 23). Another lactoferrin-derived peptide is lactoferrampin (AA 265–284 in bovines; AA 269–285 in humans) and also this peptide has a greater antibacterial activity than its native form (23–25). Efforts to further increase the antimicrobial activities of these LF-derived peptides have focused on the synthetic design of similar peptides or fusion products. For instance, LFchimera is a fusion peptide combining the synthetic peptides Lfcin 17–30 and Lfampin 265–284 from bovines, displaying a greater antibacterial activity than its separate components. The bactericidal activity of LFchimera is described against various pathogens, including *Vibrio parahaemolyticus*, *S. aureus*, *Pseudomonas aeruginosa*, *Streptococcus pneumoniae*, and *E. coli* (26–29).

LF not only decreases bacterial growth and eradicates bacteria but also impacts bacterial virulence. Bacterial pathogens have developed various mechanisms to effectively infect and invade host cells such as adherence, colonisation, invasion and biofilm formation, often aided by virulence factors. Additionally, bacterial pathogens produce proteases and toxins, which also contribute to their virulence. LF has been shown to interfere with one or more of these processes in various bacterial pathogens, thereby effectively reducing their virulence. To effectively colonise the host, one key step is the adherence of the pathogen to the host cells. LF can interfere with the adherence of various pathogens such as *Actinobacillus pleuropneumoniae* and several *E. coli* pathotypes (enteroaggregative, enteropathogenic and enterotoxigenic *E. coli*) to host cells (15, 30–33). Furthermore, it can also affect the invasiveness of *Shigella flexneri* in HeLa cells (34). By interfering with adherence and invasiveness, LF can diminish the virulence of these pathogens. Some pathogens can form biofilms, permitting them to persist at the site of infection. LF can decrease biofilm formation in various pathogens such as *Streptococcus mutans*, *A. pleuropneumoniae*, *Porphorymonas gingivalis*, *P. aeruginosa*, and *V. parahaemolyticus*, making it harder for bacteria to persist and therefore, LF aids in weakening the virulence of these pathogens (29, 30, 35–37). When host sites are already colonised by pathogenic bacteria, these can release toxins, proteases or other virulence factors, further contributing to bacterial virulence. Also here, LF impacts pathogen virulence as it can decrease the release of these virulence factors or even degrade them. For instance, LF can decrease the production of Shiga toxin 2 (Stx2) of Enterohaemorrhagic *E. coli* (EHEC) and proteases of *Mannheimia haemolytica* (38, 39). Additionally, LF has been shown to inhibit the activity of secreted proteases, such as RgpB of *P. gingivalis* and proteases of *A. pleuropneumoniae* (30, 36).

LF not only displays antibacterial effects, it also possesses antiparasitic and antiviral effects. LF exhibits antiparasitic activity against several parasites including *Entamoeba histolytica*, *Giardia lamblia*, and *Cryptosporidium parvum* (40–42). While the exact mechanism is not always clear, LF often interferes with the parasite’s iron acquisition. However, in some cases, such as with *Plasmodium falciparum*, LF can also interfere with adherence, possibly reducing its virulence (43). Although LF has been shown to interact with various parasites, its proteolytic activity has not yet been shown to play a role. Nevertheless, since many parasites possess virulence factors needed for adhesion and/or invasion, it is plausible that LF could impede parasitic virulence by interacting with these factors (44).

LF exhibits antiviral activities against several viruses including rotavirus, herpes simplex virus 1 (HSV-1), human immunodeficiency virus (HIV), hepatitis C virus (HCV), SARS-CoV-2, etc. (45–49). Unlike its antiparasitic activities, iron acquisition is less critical in LF’s antiviral mechanism. LF primarily affects viral infection by inhibiting viral entry, either by binding to host cell receptors or by binding to viral particles (50). The latter has already been shown in different viruses. For example in HCV, LF was shown to bind several cell envelope proteins, but also in HIV, where it is thought to interact with the gp120 protein and in influenza A virus, where it interacts with hemagglutinin (46, 51, 52). Although the protease activity of LF in the context of its antiviral properties has not yet been explored, the fact that LF can directly interact with virus particles suggests that its proteolytic activity could play a role.

These findings indicate that LF could interfere with multiple virulence mechanisms of pathogens. However, the underlying mechanisms by which LF affects these processes are not always clear and could involve the proteolytic degradation of virulence factors by LF.

Several studies involving LF’s antibacterial activity have already pointed out the proteolytic activity of LF. LF has been shown to directly degrade bacterial virulence factors by proteolysis but could also induce degradation of these factors through its proteolytic activity. As mentioned above, LF’s proteolytic activity could be responsible for inhibiting bacterial attachment to host cells, cellular invasion, biofilm formation and in general for decreasing bacterial pathogenicity (15, 32, 34, 53–55). As the proteolytic activity of LF, leading to the degradation of bacterial virulence factors, can contribute to its antibacterial activity but is often overlooked, this review aims to provide a comprehensive overview of the current knowledge on the proteolytic activity of LF.

## 2 Serine protease activity of LF

### 2.1 A catalytic dyad?

The antibacterial activity of LF is one of its key attributes, as it can directly act on bacteria in both a bactericidal and a bacteriostatic manner. The degradation of bacterial virulence factors by LF’s proteolytic activity contributes to this antibacterial activity, possibly decreasing the pathogenicity of these bacteria (11, 32, 53). The proteolysis of virulence factors is often a direct consequence of the serine protease activity of LF, which was first reported in 1998, in a study showing that human LF (hLF) can cleave the IgA1 protease precursor protein and the Hap adhesin of *H. influenzae* (16, 56). These bacterial virulence factors, both members of a Gram– autotransporter family, presumably facilitate the colonisation of the nasopharynx by *H. influenzae*, causing otitis media in children (16, 56). This study demonstrated that the proteolytic activity of hLF resides in its N-lobe by showing degradation of the IgA1 protease preprotein and Hap adhesin not only by full-length hLF, but also by a recombinant N-lobe of hLF. In addition, the irreversible broad-spectrum serine protease inhibitors diisopropyl fluorophosphate, phenylmethylsulfonyl fluoride (PMSF) and Pefabloc blocked this protease activity, suggesting that hLF possesses a serine protease activity (16, 53). Similar to hLF, bovine LF (bLF) was also shown to possess a serine protease activity as its degradation of the synthetic substrate N-α-benzyloxycarbonyl-Phe-Arg-7-amido-4-methyl-coumarin was almost completely inhibited by PMSF or Pefabloc (54).

Serine proteases can be divided into 13 different clans based on the structural similarity and catalytic mechanism by which they cleave peptide bonds in a wide range of proteins. These clans all possess a serine residue in their catalytically active site, often combined with a histidine and an aspartic acid residue (57). In this catalytic triad, the histidine abstracts the proton from the serine hydroxyl side chain, acting as a general base. The serine residue then initiates a nucleophilic attack on a carbonyl group of an amide bond within a protein substrate. The third residue, aspartic acid, assists in correctly orienting the histidine and neutralises the charged histidine intermediate form. Although most serine proteases possess a catalytic triad, some possess a catalytic dyad, consisting of a serine and a lysine, such as the mitochondrial signal peptidase I and several bacterial proteases. Here, the lysine residue acts as a general base, as its Ɛ-amino group can increase the nucleophilicity of the serine, followed again by nucleophilic attack on a protein substrate (58).

LF is thought to be such a non-canonical serine protease with a catalytic dyad, as its primary amino acid structure lacks features of a typical serine protease. After showing the serine protease activity within the N-lobe of hLF, the crystal structure of hLF was examined in an attempt to identify the serine residues involved in its protease activity (53). Here, Ser259 and Lys73 emerged as potential candidates responsible for the protease activity of hLF. These residues project into the large cleft between the N-and C-lobes of hLF, possibly accommodating large substrates, where these undergo proteolytic cleavage. Using site-directed mutagenesis, Ser259 or Lys73, both present in the N-lobe of hLF, were each replaced by an alanine residue. A control where Pro251, a residue relatively remote from the putative active site, was mutated to a valine residue was included to ensure that mutation did not affect the proteolytic activity of hLF. Then, the proteolytic activity towards the *H. influenzae* IgA1 protease preprotein and the Hap adhesin was assessed (53). Whereas mutation of Pro251 did not influence the proteolytic activity of hLF, mutation of Ser259 or Lys73 to Ala completely abrogated the ability of the N-lobe of hLF to cleave the IgA1 protease preprotein (53). Nevertheless, only the Ser259 mutation abolished the degradation of the Hap adhesin by hLF, whereas the Lys73 mutation resulted only in partial degradation of this protein (53). This shows that, most likely, Ser259 and Lys73 could form a catalytic dyad accounting for the serine protease activity of hLF. The authors speculate that the Lys73 residue could act as a general base, which is deemed possible as the neighbouring amino acids inside the LF structure could provide a favourable environment to keep this residue in the necessary unprotonated state. Alternatively, a high density of Arg residues in the substrate could also keep Lys73 in the unprotonated state (53). After positioning of Lys73 close to the O-atom of Ser259 using standard rotamers, this could lead to hydrogen binding of Lys73 to Ser259, ultimately resulting in a nucleophilic attack of Ser259 on a protein substrate (53).

Later, another study attempted to identify the amino acid residues involved in hLF’s serine protease activity, while also verifying the conservation of these amino acids in other mammalian LFs from bovine, buffalo and horse (54). Aligning the amino acid sequences of the N-lobes of LF from these species showed 12 conserved Ser residues. To further identify possible candidates involved in the serine protease activity of LF, pK_a_ shift calculations were conducted, as the polarisation of the hydroxyl group of Ser is required for its proteolytic function. This analysis showed that only three Ser residues (Ser12, Ser184, and Ser283) displayed a substantial pK_a_-shift, resulting in strong nucleophilicity of these residues, and thus marks them as potentially interesting candidates (Figure 1) (54). Remarkably, Ser259 was no longer considered a potential candidate in the catalytic site responsible for the protease activity of LF (Figure 1) (54). Although the serine protease active site of LF could be different depending on the species, we consider this possibility rather unlikely as the sequence similarity of LF among species is quite high. To reinforce the hypothesis that the same active site could be present in different mammalian LFs, mutagenesis studies similar to Hendrixson et al. should be performed in other LFs such as bLF or porcine LF (pLF) (53).

There is not only disagreement regarding the amino acids involved in the serine protease catalytic region of LF, but also on the protein substrate accommodation in relation to iron saturation of the LF molecule. Hendrixson et al. found that hLF’s serine protease activity is independent of the iron saturation level of LF, as the proposed catalytic region is remote from the iron-binding site of the N-lobe of hLF (53). On the contrary, Masucci et al. showed that iron binding (as well as lipopolysaccharide binding) inhibits bLF’s serine protease activity. Furthermore, only 10% of the bLF molecules showed proteolytic activity, and no conformational changes were seen when comparing proteolytically active and inactive bLF (54).

### 2.2 Substrate specificity of LFs

**Figure 2:**
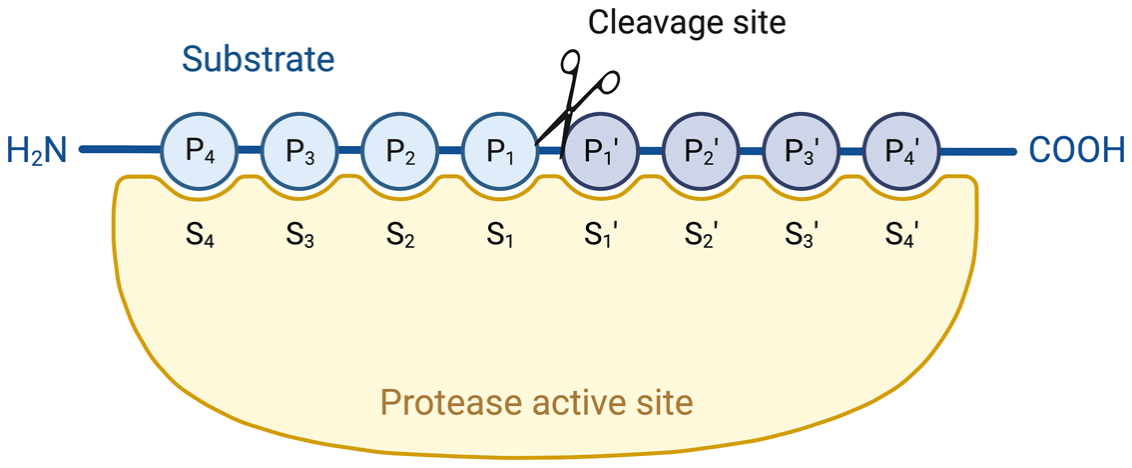
Serine protease active site interacting with a peptide substrate. Protease binding sites (labelled S1, S1′, etc.) are complementary to amino acid residues in the substrate (labelled P1, P1′ etc.). Cleavage occurs at the bond between the P1 and P1′ residues. Created with Biorender.com.

When taking a closer look at the interaction of a substrate with most of the serine proteases, the amino acid residues adjacent to the cleavage site often determine their substrate specificity. Typically, the protein substrate is first accommodated in the substrate recognition domain of the serine protease. Next, the substrate binds to residues in the binding pocket using a hydrogen atom on its side chain. However, hydrophobic interactions and electrostatic interactions can also play a role here, depending on the serine protease and the preferred substrate (59). The binding sites of the serine protease N-terminal to the cleavage site are termed S1, S2, …, whereas those located C-terminal to the cleavage site are termed S1′, S2′, … (Figure 2). Similarly, the amino acid residues of the substrate interacting with these binding sites are termed P1, P2, … (N-terminal to the cleaved bond) and P1′, P2′, … (C-terminal) (60). The chemically and spatially complementary interaction between P1 and S1 largely determines the substrate specificity of most serine proteases. Trypsin-like serine proteases, for example, prefer Arg/Lys residues at P1, whereas chymotrypsin-like serine proteases prefer hydrophobic amino acid residues such as Phe at P1 (60, 61).

As LF is an atypical serine protease, less is known about its substrate specificity and a substrate cleavage site has not yet been identified. Previous work showed that hLF can cleave both the IgA1 protease preprotein and Hap adhesin of *H. influenzae* at Arg-rich regions, being VRSRRAAR and RRSRRSVR, respectively. More precisely, these proteins were cleaved after the sequence RSRR or RRSR. This cleavage site is similar to that of certain Ca^2+^-dependent endoproteases (53). However, no such Arg-rich regions can be found in the sequence of EspB, a bacterial protein of the type III secretion system (T3SS) of enteropathogenic *E. coli* (EPEC), which was also proteolytically degraded by hLF (62). Moreover, these Arg-rich regions are also absent in other bacterial proteins degraded by bLF and pLF (15). This suggests that LFs from various mammalian species do not all specifically target Arg-rich regions when degrading bacterial proteins by their serine protease activity but rather act in a species-dependent manner, meaning that bLF, pLF and hLF all have their own specific target regions. However, this seems rather unlikely, as proteins such as EspB and the IgA1 protease preprotein are both degraded by the same LF, i.e., hLF, but do not have similar Arg-rich regions that could be targeted by hLF (62). Another possibility is that LFs from different origins could degrade bacterial proteins derived from the same pathogen in a similar fashion, targeting a species-specific sequence on the substrate level. In this context, our research group showed that both bLF and pLF degraded several bacterial virulence factors from porcine enterotoxigenic *E. coli* (ETEC), however, no sequence could be identified as a common target for both bLF and pLF (15). Altogether, it appears that LFs of different species can target different sites and that more research is needed to identify the specific target sequences of LF.

## 3 Degradation of bacterial virulence factors by LF

LF’s serine protease activity may be less well-studied than its other activities, however it might significantly contribute to its antibacterial function. So far, LF has been shown to degrade various bacterial virulence factors or is thought to be indirectly involved in the degradation of bacterial proteins through its proteolytic activity. Degradation of these virulence factors could result in a lower adherence to host cells, decreased pathogen motility or decreased invasion of host cells by the bacteria, all of which may contribute to reduced pathogenicity of these bacteria towards the host (11, 32, 53).

### 3.1 LFs degrade various bacterial virulence proteins via their serine protease activity

LF’s serine protease activity causes the breakdown of several bacterial virulence factors, possibly leading to a decreased pathogenicity of several bacterial organisms. The serine protease activity was first reported in bLF and hLF, and later in pLF (15, 16, 54). Whereas the first study reporting bLF’s serine protease activity only addressed the degradation of a synthetic substrate, i.e., N-α-benzyloxycarbonyl-Phe-Arg-7-amido-4-methyl-coumarin, the first report concerning the serine protease activity of hLF already showed degradation of bacterial virulence factors (15, 16, 54). Hendrixson et al. showed that the serine protease activity, residing in the N-lobe of hLF, caused the release and subsequent degradation of the IgA1 protease preprotein and direct degradation of Hap adhesin (53). These proteins are both bacterial virulence factors involved in the pathogenesis of *H. influenzae*, causing respiratory tract diseases, such as otitis media, bronchitis and pneumonia, but also systemic diseases such as meningitis and epiglottis, mostly in children. Degradation was shown by incubation of whole bacterial cell lysates with the N-lobe of hLF. Furthermore, when inactivating hLF using the serine protease inhibitors diisopropyl fluorophosphate, PMSF and Pefabloc, no degradation of bacterial virulence factors was observed, confirming hLF’s serine protease activity (16, 53). Later, a study also assessed the serine protease activity of hLF towards a virulence factor of *Neisseria meningitidis*, another Gram– pathogen causing (meningococcal) meningitis. Here, hLF could proteolytically cleave the Neisserial Heparin-binding antigen (NHBA), a virulence factor which is thought to be involved in various steps of meningococcal pathogenesis (63, 64). The serine protease activity of hLF was confirmed using the serine protease inhibitor PMSF (63). Interestingly, this protein was cleaved immediately downstream of an Arg-rich region within this virulence factor, in contrast to earlier findings suggesting that hLF cleaves bacterial virulence factors targeting an Arg-rich region (53, 63, 64).

LF can also degrade other virulence factors important in bacterial pathogenesis, such as proteins of the T3SS (32, 62, 65). Bacteria employ this system to directly inject bacterial proteins into host cells across both bacterial and host membranes. The T3SS is found across several Gram– pathogens, such as *Salmonella*, *C. psittaci*, *Pseudomonas* spp., EHEC, and EPEC (20, 32, 62, 65). In enteric pathogens, such as EHEC, which causes haemorrhagic colitis/haemorrhagic uremic syndrome, and EPEC, causing infant diarrhoea in humans, the T3SS plays an important role (66). After initial adherence of EPEC/EHEC to host intestinal epithelial cells, the T3SS, mainly composed of the proteins EspA, EspB and EspD, forms a stable needle complex with other proteins at the bacterial surface. EspA forms a tube between the bacterium and the host cell via which EspB and EspD are delivered to the host cell. There, EspB and EspD form a pore in the host cell membrane allowing bacterial proteins to be transferred directly into these cells (32, 65). After delivery of the effector protein Tir (translocated intimin receptor) to the host, the bacterial outer membrane protein intimin binds to Tir and allows intimate bacterial attachment (67). This leads to the activation of cell signalling pathways, resulting in actin polymerisation and loss of microvilli, ultimately culminating in the formation of the typical ‘pedestal’ or the ‘attaching and effacing’ lesion (67–70). Moreover, in EPEC, the T3SS proteins EspA, EspB, and EspD are shown to mediate lysis of red blood cells (71, 72). hLF, in non-growth-inhibiting concentrations, has been shown to inhibit both EPEC adherence to HEp-2 cells and EHEC adherence to Caco-2 cells (32, 62, 65). Furthermore, hLF inhibited the induction of actin polymerisation in HEp-2 cells and blocked EPEC-induced hemolysis (32, 62). To investigate whether an effect of hLF on the T3SS of EPEC was involved herein, cell-associated levels of EspA, EspB, and EspD in bacterial pellets were evaluated every hour after incubation with hLF for 7 h. hLF decreased cell-associated levels of EspA, EspB, and EspD with EspB showing the most drastic reduction (32, 62). This suggests that hLF induces the fast release of these virulence factors from bacterial cells, which could be followed by proteolytic digestion. Furthermore, it was shown that hLF degrades purified EspB from EPEC via its serine protease activity, since the serine protease inhibitors antipain, chymostatin and soybean trypsin inhibitor completely inhibited EspB degradation by hLF (32, 62). Similarly, EspB degradation was observed for both hLF and bLF in EHEC, but the serine protease activity of LF was not assessed (65). Degradation of EspA and EspD, however, was not explored. Altogether, the interference of hLF and bLF with the EspA, EspB, and EspD proteins of the T3SS, potentially blocking crucial steps in EPEC/EHEC pathogenesis, suggests that hLF and bLF may confer protection against these enteric pathogens, although further research is advised (32, 62). A two-step mechanism was suggested for hLF to disarm the T3SS of EPEC. In the first step, hLF could bind to the lipid A part of lipopolysaccharides, causing destabilisation of the bacterial cell wall, potentially resulting in the release of proteins anchored in the bacterial outer membrane, such as the T3SS (32, 62). Secondly, the digestion of the bacterial proteins by hLF is involved as it was shown that hLF degrades EspB. While hLF exhibited a less pronounced reduction in cell-associated EspA and EspD compared to EspB, prompting the authors to solely evaluate the degradation of EspB, it may still be worthwhile to investigate the degradation of EspA and EspD in EPEC. Especially EspA could be interesting as quite a clear decrease in cell-associated EspA could be observed and EspA was shown to be degraded in EHEC by both hLF and bLF (65).

LF not only impairs bacterial virulence by disrupting the T3SS, it also affects other surface-associated virulence factors. Both bLF and pLF can degrade specific adhesins, termed fimbriae or colonisation factors, which play a crucial role in the pathogenesis of porcine ETEC and Shiga toxin-producing *E. coli* (STEC) strains (15). These porcine pathogens cause diarrhoea and oedema disease, respectively, with bacterial adhesion to the gut epithelium, mediated by its fimbriae, being a crucial step for disease onset (73). In the pig, pathogenic *E. coli* strains producing F4 and F18 fimbriae are most prevalent (73). Both F4 and F18 fimbriae are composed of several subunits. We recently showed that, using non-growth inhibiting concentrations, both bLF and pLF can degrade the major subunit of F4 fimbriae, the adhesin FaeG (15). Degradation of this subunit was confirmed for the different subtypes of F4 fimbriae (F4ab, F4ac, F4ad), each showing variation in the FaeG amino acid sequence, resulting in binding of different F4 receptors (74). Moreover, bLF and pLF could also degrade the major subunit of F18 fimbriae, FedA, and the tip adhesin FedF (15). As these fimbriae are essential for bacterial attachment to host cells, adhesion assays were conducted, using LF-treated ETEC strains, on both IPEC-J2 cells (for F4-fimbriated ETEC strains) and isolated porcine villi (for F18-fimbriated ETEC strains) (15). These bLF-and pLF-treated ETEC strains showed a decreased adhesion to the gut epithelial cells and inhibiting the serine protease activity of bLF and pLF completely abolished this reduced adhesion (15). In this study, the influence of bLF and pLF on the degradation of fimbriae and ETEC adherence was only assessed using a selection of F4^+^ and F18^+^ fimbriae porcine ETEC strains (15). To further validate these findings, it would be interesting to repeat these experiments using other types of porcine fimbriae or even human fimbriae. In addition to fimbriae, bLF and pLF were shown to degrade flagellin from ETEC, the major component of bacterial flagella, which control bacterial motility. However, it is unknown whether bLF or pLF can also degrade flagellin of other bacterial origin, such as *Salmonella*. The above mentioned *in vitro* experiments indicate the potential of LF as an alternative treatment for post-weaning diarrhoea in piglets. Our follow-up *in vivo* studies supported these promising results, as we showed that both bLF and pLF reduced ETEC bacterial motility and bacterial adhesion of an F18^+^ ETEC strain to small intestinal epithelial cells, by degradation of flagellin and F18 fimbriae, respectively (75). Moreover, we showed that bLF and pLF reduced the ETEC-induced fluid secretion in a small intestinal segment perfusion model (75). However, when bLF was orally administered to post-weaning piglets in a challenge *in vivo* experiment, no effects were seen on faecal excretion of an F18^+^ STEC strain, but the specific F18-specific IgG serum response was abolished (76).

Aside from the proteolytic effects that LF displays on several membrane-associated proteins such as the T3SS, fimbriae and flagellin, LF also shows a proteolytic effect on secreted proteins such as toxins. Toxins are often crucial in the pathogenesis of different enteric pathogens such as EHEC/ETEC/STEC. Toxins can cause fluid secretion leading to diarrhoea, but also cell apoptosis, potentially leading to organ damage (77–79). The most important toxins of the aforementioned pathogenic *E. coli* subtypes are Shiga toxins 1 and 2 (Stx1 and Stx2) for EHEC and STEC, Stx2e for porcine STEC strains and the heat-labile enterotoxin (LT) for ETEC (73, 78, 80). These toxins all belong to the AB_5_ toxin family, which is characterised by five identical B subunits, responsible for cellular binding, and one A subunit, containing the catalytic domain (78). Research showed that bLF can cleave the B subunit of Shiga toxin 2 secreted by the EHEC O157:H7 strain, but not the A subunit (38). Furthermore, the degradation of the LT toxin by bLF and pLF was also assessed, but no degradation was observed (15). Further research should be conducted to test whether B subunits of other Shiga toxins are also degraded and whether LF is capable of degrading other bacterial toxins, such as the heat-stable enterotoxins secreted by ETEC, STa and STb.

The above mentioned studies have clearly shown that LF can degrade a variety of bacterial virulence factors by its serine protease activity (Table 1). Together with its iron binding and bactericidal activity, this protease activity plays a role in reducing the colonisation and pathogenicity of the investigated bacteria.

**Table 1:** Overview of bacterial virulence factors degraded by the serine protease activity of LF.

Bacteria	Virulence factor	Function	LF origin	Effect	Ref.
*Haemophilus influenzae*	Hap adhesin (AT protein)	Promotion of intimate interaction with human epithelium	(Recombinant) apo-hLF	Decreased adherence to Chang cells	(16)
	IgA1 protease preprotein (AT protein)	Cleavage and inactivation of human IgA1	(Recombinant) apo-hLF	Unknown	(16)
*Neisseria meningitidis*	NHBA	Bacterial adhesion to epithelial cells, biofilm formation, bacterial survival, vascular leakage	hLF	Unknown	(63, 64)
EPEC	EspB (T3SS protein)	Attaching and effacing (A/E) lesion formation	(Recombinant) apo-hLF	Blocking of EPEC adherence to HEp-2 cells Blocking of EPEC-induced hemolysis Induction of EPEC actin polymerisation in HEp-2 cells	(32)
EHEC	EspAEspB(T3SS protein)	Attaching and effacing (A/E) lesion formation	holo-bLF, holo-hLF	Inhibition of EHEC attachment to Caco-2 cells	(65)
ETEC	FaeG	Adherence to small intestinal enterocytes	Apo-bLF, (recombinant) apo-pLF	Decreased ETEC adherence to IPEC-J2 cells	(15)
ETEC/STEC	FedAFedF	Adherence to small intestinal enterocytes	Apo-bLF, (recombinant) apo-pLF	Decreased ETEC/STEC adherence to isolated porcine villi	(15)
ETEC	Flagellin	Motility of the pathogen	Apo-bLF, (recombinant) apo-pLF	Decreased ETEC motility	(15)
EHEC	Stx2 receptor-binding B subunit	Binding to cellular Stx2 receptor	Apo-bLF	Potential prevention of Stx2 binding to cellular receptor and subsequent internalisation	(38)

AT, autotransporter; bLF, bovine lactoferrin; EHEC, Enterohaemorrhagic *Escherichia coli*; EPEC, Enteropathogenic *Escherichia coli*; ETEC, Enterotoxigenic *Escherichia coli*; hLF, human lactoferrin; NHBA, Neisserial Heparin Binding Antigen; pLF, porcine lactoferrin; Stx2, Shiga toxin 2; STEC, Shiga toxin-producing *Escherichia coli*; T3SS, Type III secretion system.

While LF can degrade virulence factors from different pathogens, its protease activity cannot be generalised towards all pathogens or virulence factors. Indeed, as summarised in Table 2, several other bacterial virulence factors could not be degraded by LF (Table 2).

**Table 2:** Overview of bacterial virulence factors not degraded by the serine protease activity of LF.

Bacteria	Virulence factor	Function	LF origin	Ref.
*Haemophilus influenzae*	P2 P5 P6	Contribute to bacterial outer membrane stability Affect membrane protein composition crucial for interaction with the human host	(Recombinant) apo-hLF	(16, 81)
EHEC	Intimin (T3SS protein)	Attaching and effacing (A/E) lesion formation	(Recombinant) apo-hLF, holo-hLF, holo-bLF	(65, 82)
EHEC	Stx2 receptor-binding A subunit	Binding to eukaryotic ribosome Blocking of protein synthesis in target cells (after cleavage into A1 and A2 fragments)	Apo-bLF	(38, 83)
ETEC	Heat-labile enterotoxin (LT)	Disrupt electrolyte homeostasis, resulting in fluid loss and diarrhoea	Apo-bLF, (recombinant) apo-pLF	(15, 84)

A/E; attaching and effacing; bLF, bovine lactoferrin; EHEC, Enterohaemorrhagic *Escherichia coli*; ETEC, Enterotoxigenic *Escherichia coli*; hLF, human lactoferrin; LT, heat-labile enterotoxin; pLF, porcine lactoferrin; STEC, Stx2, Shiga toxin 2, Shiga toxin-producing *Escherichia coli*; T3SS, Type III secretion system.

### 3.2 The potential role of LF’s proteolytic activity in the reduction of bacterial virulence

As mentioned above, LFs can degrade many bacterial proteins via their serine protease activity. This was investigated using purified LFs and bacterial virulence factors as well as specific serine protease inhibitors. Besides the abovementioned studies, other studies report the degradation of bacterial virulence factors, possibly by LFs or by other proteases activated through LFs (Table 3). Whether the serine protease activity of LFs is involved here, is not yet known.

**Table 3:** Overview of other bacterial virulence factors that may be degraded as a direct or indirect consequence of the proteolytic ACT of LF.

Bacteria	Virulence factor	Function	LF origin	Effect	Ref.
*Actinobacillus actinomycetemcomitans*	Aae (AT protein)	Adhesion to epithelial cells	hLF	Decreased *Actinobacillus actinomycetemcomitans* adherence to KB cells	(85)
EAEC	Aggregative adherence fimbriae (AAF) (adhesin)	Adherence to intestinal mucosa	Apo-bLF, holo-bLF	Inhibition of EAEC adherence to HEp-2 cells Inhibition of EAEC biofilm formation	(31)
*Shigella flexneri*	IpaB IpaC (invasion antigens)	Invasion of bacteria in epithelial cells	(Recombinant) apo-hLF, (recombinant) holo-hLF	Decreased invasion in HeLa cells	(34)
*Chlamydia psittaci*	CopB CopD (T3SS protein)	Pore formation allowing transfer of bacterial effector proteins to host cells	apo-hLF, apo-bLF	Unknown	(20, 86)
*Yersinia enterocolitica* *Yersinia tuberculosis*	Invasin (adhesin)	Binding and internalisation into mammalian cells	bLFcin	Inhibition of adhesion of *Yersinia enterocolitica* and *Yersinia tuberculosis* to HEp-2 cells	(87)
*Pseudomonas aeruginosa*	Elastase	Degrades elastin causing damage in pulmonary tissue and blood vessels	bLF, bLFcin, bLFampin, bLFchimera	Unknown	(29)
	Pyocyanin	Causes damage to ciliary function, epidermal cell growth Inhibits cell respiration	bLF, bLFcin, bLFampin, bLFchimera	Unknown	(29)

AAF, aggregative adherence fimbriae; AT, autotransporter; bLF, bovine lactoferrin; EAEC, Enteroaggregative *Escherichia coli*; hLF, human lactoferrin; T3SS, Type III secretion system.

For instance, some proteins showing a high degree of homology to the Hap adhesin and IgA1 protease preprotein from *H. influenzae*, both cleaved by hLF, could potentially be cleaved by hLF as well. These homologous proteins are found in several bacterial organisms such as *S. pneumoniae*, *Neisseria gonorrhoea*, and *Actinobacillus actinomycetemcomitans* (16). For *S. pneumoniae* and *N. gonorrhoea*, no research has yet been performed concerning the proteolytic activity of hLF on similar autotransporter proteins. However, for *A. actinomycetemcomitans*, a pathobiont in periodontitis, the autotransporter Aae, involved in the adhesion of *A. actinomycetemcomitans* to epithelial cells, is cleaved by human milk whey (containing LF at 0.5 mg/mL) (85). This was observed upon western blot analysis of cell pellets and supernatants, which were obtained after treatment of *A. actinomycetemcomitans* (10^7^ cells) with human milk whey (85). Incubating *A. actinomycetemcomitans* with human milk whey not only resulted in cleavage of Aae, but also resulted in a decreased adhesion of the bacteria to epithelial cells (85). While this study hints at a potential degradation of Aae by hLF, further research using purified hLF, Aae and inactivated hLF should be performed to exclude other possibilities.

As mentioned earlier, bLF and pLF can degrade colonisation factors from porcine ETEC or STEC strains. Another type of fimbriae that are degraded by bLF, are the aggregative adherence fimbriae (AAF) of enteroaggregative *E. coli* (EAEC), a pathogen causing diarrhoea in children and travellers (31). AAFs are essential for adherence of EAEC to the intestinal mucosa. After incubation of EAEC with bLF under non-bacteriostatic conditions, western blot analysis of bacterial cell pellets and supernatants revealed that bLF induced the release and degradation of cell-associated AAF. In addition, bLF decreased adherence of EAEC to HEp-2 cells, inhibited EAEC biofilm formation and increased EAEC autoagglutination, independent of its iron saturation level (31). This shows that bLF could potentially decrease EAEC attachment and colonisation in the host. The release and subsequent degradation of AAF by bLF is hypothesised to be caused by the interaction of LF with the bacterial membrane, destabilising several surface-anchored bacterial proteins. Another possibility is that bLF can directly degrade AAF. However, this requires further research using purified AAF and serine protease inhibitors.

LF is not only involved in the degradation of *E. coli* virulence factors, but can also induce degradation of virulence factors from other enteric pathogens, such as *S. flexneri*. hLF was shown to induce degradation of the invasion antigens IpaB and IpaC of *S. flexneri*, which reduced the invasion of *S. flexneri* into HeLa cells (34). Both IpaB and IpaC are important virulence factors secreted as a complex (IpaBC) in response to contact with eukaryotic cells and are required for invasion of mammalian host cells by *Shigella* (34). Upon incubation of *S. flexneri* with various concentrations of hLF, IpaB and, to a lesser extent IpaC, were released from the bacterial cell surface and subsequently degraded. The degradation of cell-associated IpaB and IpaC in the presence of *S. flexneri* was partially blocked by some serine protease inhibitors, such as antipain, chymostatin and soybean trypsin inhibitor but not by benzamidine (34). While this seems to indicate that hLF can degrade these proteins, hLF was unable to degrade purified IpaB and IpaC. This implies that hLF did not have a direct proteolytic effect on these virulence factors and could only play an indirect role in the degradation of these proteins (34). The authors hypothesised that hLF might disrupt the bacterial cell membrane, leading to an increased secretion of the invasion proteins and thus exposing the IpaBC complex to bacterial outer membrane proteases (34). Other research showed that hLF and bLF inhibited the infection of *C. psittaci*, an avian intracellular pathogen, in chicken macrophages, with the authors speculating that LF’s serine protease activity could be responsible for this, by degrading CopB and CopD, proteins of the chlamydial T3SS, as these are structurally similar to IpaB and IpaC from *S. flexneri* (20). To elucidate whether the serine protease activity of LF is involved or a destabilisation of the bacterial membrane by LF is responsible for inhibition of *C. psittaci* infection in chicken macrophages, further research is needed (20).

Lastly, a study showed that the serine protease activity of hLF plays a crucial role in the bactericidal action against *S. aureus*. Remarkably, the bactericidal activity of hLF towards *S. aureus* was completely abolished when the proteolytic activity of hLF was inhibited using PMSF. Unfortunately, here, degradation of *S. aureus* virulence factors was not assessed (88).

In literature, not only the effects of LF on bacterial virulence factors are investigated, but also the effects of LF-derived peptides, such as Lfcin or lactoferrampin, towards bacterial virulence factors are explored, although less frequently. In a study assessing the effects of both the LF-derived peptide bLFcin and bLF on *Yersinia enterocolitica* and *Yersinia pseudotuberculosis*, only bLfcin seemed to affect the bacterial virulence factor invasin, however, the precise mechanism is unclear. bLFcin, used in a non-growth inhibiting concentration of 0.5 mg/mL, inhibited the adhesion of *Yersinia enterocolitica* and *Yersinia pseudotuberculosis* to HEp-2 cells. However, this was only observed when the invasin virulence factor showed a high expression (87). Invasin is a non-pilus-associated adhesin, anchored to the bacterial outer membrane, and is the most important virulence factor in the binding and internalisation process of *Yersinia pseudotuberculosis* into mammalian epithelial cells. Surprisingly, no effect of bLFcin on adhesion and invasion was observed when invasin expression levels were low. In conclusion, bLFcin seems to affect invasin, however, the mechanism is unclear (87). The bLF-derived peptides bLFcin, but also bovine lactoferrampin and LFchimera might affect other virulence factors as well. It was shown that these antimicrobial peptides inhibited the production of elastase and pyocyanin, virulence factors of the opportunistic pathogen *P. aeruginosa*. While elastase damages pulmonary tissue and blood vessels by degrading elastin, the secondary metabolite pyocyanin inhibits ciliary function, epidermal cell growth and cell respiration. Currently, it is unknown how these bLF-derived peptides interfere with the production of these virulence factors (29).

## 4 Effect of LF on bacterial adherence and invasion

Two crucial steps involved in bacterial colonisation are (i) adherence to host cells and (ii) invasion of host cells. As mentioned above, LF can induce degradation of virulence factors which aid in bacterial adherence and invasion, possibly leading to decreased pathogenicity of some bacterial organisms. However, LF could also affect bacterial adherence to or invasion of host cells, without proof of the degradation of any virulence factors.

A lot of research has already been performed regarding the anti-adhesive properties of LF and a substantial part of this research focuses on the interaction of LF with enteric pathogens, such as the different *E. coli* pathotypes. To this end, researchers have shown that both hLF and bLF block the attachment of EHEC/STEC to HEp-2 cells (33). Furthermore, another diarrheagenic pathotype of *E. coli*, EPEC, upon incubation with hLF, showed a decreased adhesion to HeLa cells (89). Another study showed that not only LF, but also the fusion peptide LFchimera, consisting of LFcin17–30 and LFampin265–284 could decrease adherence of EPEC to HEp-2 cells. Furthermore, they could also decrease actin polymerisation, typically associated with EPEC infection in these cells (90). For EAEC and diffusely adhering *E. coli* (DAEC), the addition of a human milk fraction containing LF, decreased adherence to HeLa cells (91). Here, only a pooled milk fraction was tested, which not only contained hLF, but also free secretory component, serum albumin and casein, so interpretation of these results should be done carefully (91). Especially as several studies suggested that other milk components such as glycoconjugates like glycolipids, glycoproteins, mucins, glycosaminoglycans and oligosaccharides can also inhibit pathogen binding to host cells (91). Another important causative agent of diarrheal illness, *Salmonella enterica* serovar Typhimurium, also showed a decreased bacterial adhesion to HeLa cells when adding hLF, yet the involved mechanism remains unclear (92). Additionally, hLF was shown to interact with *Salmonella* proteins present in an enriched extract of fimbriae and an enriched extract of T3SS proteins using immunoblotting (92).

Other studies show that bLF can also affect bacterial adherence of other pathogens, such as bacteria involved in the pathogenesis of pneumonia. Here, bLF is shown to inhibit *A. pleuropneumoniae* adhesion to porcine buccal cells and *S. pneumoniae* adhesion to different types of cells of the upper respiratory tract (laryngeal, lung, and nasopharyngeal cells) (30, 93). Furthermore, bLF can also bind to cable pili of *Burkholderia cenocepacia*, a respiratory pathogen involved in the pathogenesis of cystic fibrosis, leading to a decreased adhesion to mucin (94). Nevertheless, the addition of bLF to *B. cenocepacia* as well as to *P. aeruginosa* did not result in an inhibited adhesion of these pathogens to the human bronchial A549 cell line, although it did significantly inhibit invasion of these pathogens into the same cell line (95). For *L. monocytogenes*, a Gram+ intracellular pathogen potentially causing severe systemic disease, a similar phenomenon was observed, as bLF was unable to decrease adhesion to both Caco-2 and HT-29 cells, although it could significantly inhibit invasion of the pathogen into these intestinal epithelial cells (96).

As LF is shown to interfere with the invasion or adherence of these bacterial pathogens, it would be interesting to investigate whether LF could degrade some of the virulence factors involved in the binding of these pathogens to host cells.

## 5 Discussion

To this day, LF is best known for its antibacterial activity against both Gram− and Gram+ bacteria. It limits bacterial growth through its iron-binding abilities and kills bacteria by binding to and disrupting their outer membrane (11, 13, 14). However, an important but often overlooked feature is the proteolytic activity of LF. The latter can impair the function of bacterial virulence factors, decreasing the ability of bacteria to adhere to or invade host cells, possibly culminating in a reduced pathogenicity of several bacterial organisms (15, 32, 34, 53–55). The proteolytic activity of lactoferrin could possibly contribute to the synergism of the action of lactoferrin and its peptides against pathogenic bacteria, enhancing their antimicrobial activity. Cleavage of bacterial surface structures by lactoferrin might allow easier access for both lactoferrin and its peptides to the bacterial membrane, making these bacteria more vulnerable to antimicrobial attack. Moreover, the cleavage of bacterial virulence factors might create or expose additional binding sites for lactoferrin and its peptides, further potentiating their antimicrobial activity.

LF is an atypical serine protease, as it contains a catalytic dyad (53). Previous research suggests that, for hLF, Ser259, and Lys73 constitute this catalytic dyad (53). However, another report suggests that the catalytic dyad could be different in other mammalian LFs, as Ser259 was suggested not to be involved in bLF’s proteolytic activity (54). Further mutational analysis of Ser and Lys residues in the N-lobe of LF should be performed to identify the active site, responsible for the serine protease activity of LF. Although different LFs display a high sequence homology (9), the catalytic domain could still differ between mammalian LFs. Besides the uncertainty concerning the amino acids that comprise the catalytic dyad, it is also unclear whether iron-binding of LF influences its proteolytic activity (53, 54). Therefore, in future research on the proteolytic activity of LF, it is important to test both apo-and holo-LF.

LF degrades multiple bacterial virulence factors, however, it is unclear whether LF targets a specific sequence within these pathogenic proteins (15, 16, 32, 62, 65, 81). One study showed that hLF targeted Arg-rich regions in bacterial virulence factors of *H. influenzae*, yet no such regions were present in EspB of EHEC, another virulence factor degraded by hLF (53, 65). Furthermore, other mammalian LFs such as pLF and bLF degraded bacterial virulence proteins not containing any Arg-specific regions (15, 53). This raises the question of whether LF recognises a specific target sequence in bacterial virulence factors and whether different LFs target the same sequence. To assess this, an extensive *in silico* sequence analysis might need to be conducted to identify potential species-specific target sequences for different LFs. Although a variety of bacterial virulence factors is degraded by LF’s serine protease activity, this field is still underexplored, as current studies often only assess the degradation of a single virulence factor from a single bacterial pathogen. Therefore, it could be important to investigate the degradation of multiple proteins by LFs within a single pathogen. For example, LF degrades EspB of EPEC, but the degradation of other proteins belonging to the T3SS, such as EspA and EspD should also be verified (54). Alternatively, LF’s proteolytic activity could be tested in similar proteins across different pathogens. For example, LF’s proteolytic activity might be tested, not only for Stx2 of EHEC, but also for other Shiga toxins (81). Moreover, degradation of autotransporter proteins of other pathogens such as *N. gonorrhoea*, *S. pneumoniae*, homologous to the Hap adhesin and IgA1 protease preprotein of *H. influenzae* by LF should be tested (16, 53). To avoid ambiguity, purified LF and purified virulence factors should be used together with serine protease inhibitors to ascertain the ability of LF to degrade these virulence factors. Although degradation of bacterial virulence factors by the serine protease activity of LF has been studied *in vitro*, *in vivo* data are currently lacking. This should be done in the future, as it could confirm whether the degradation of virulence factors leads to reduced colonisation and results in a compromised pathogenicity of bacteria.

**Figure 3:**
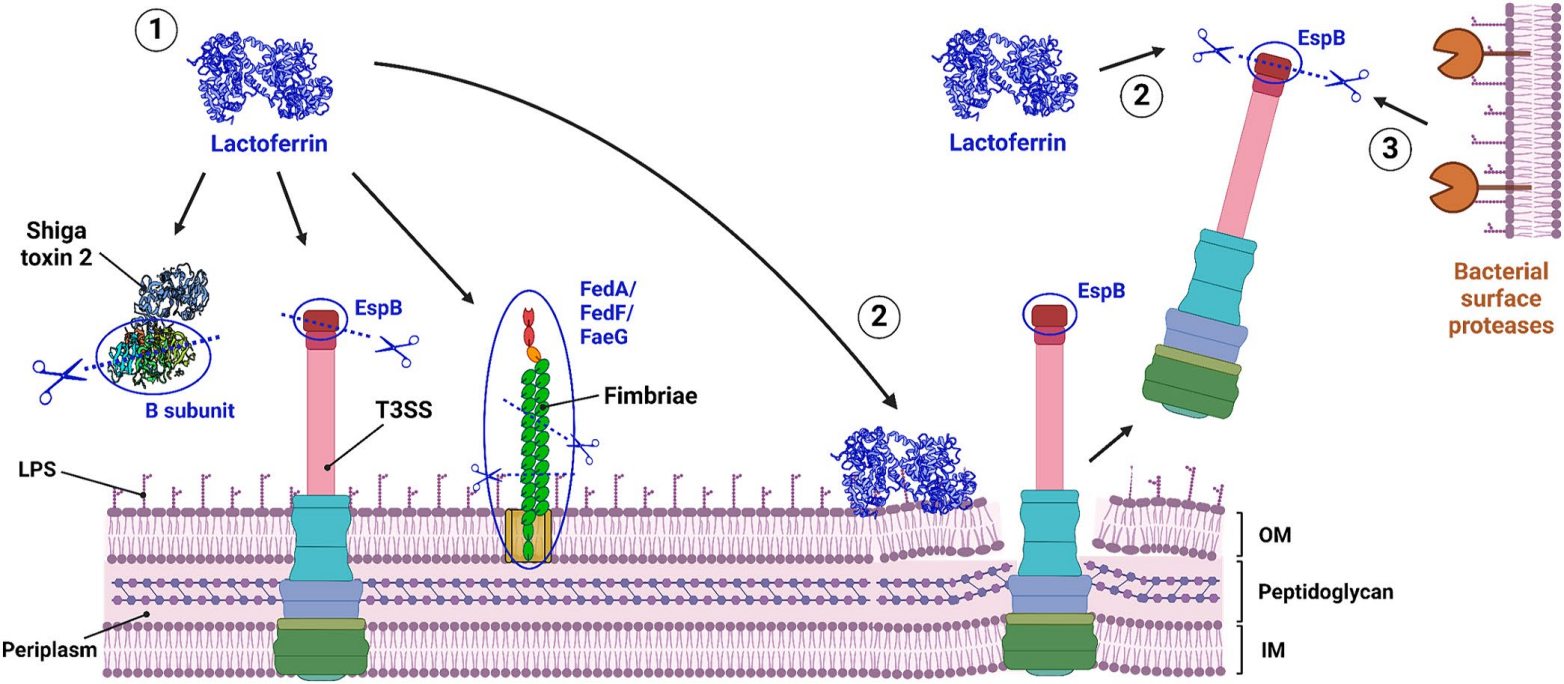
Three proposed mechanisms for the proteolytic degradation of bacterial virulence factors by lactoferrin. (1) Direct degradation, where lactoferrin directly breaks down bacterial virulence factors. (2) Indirect degradation, where lactoferrin binds to LPS structures on the outer membrane, causing membrane disruption, leading to the release of bacterial virulence factors, which LF then degrades. (3) Induced degradation, where membrane disruption by LF binding triggers the degradation of released virulence factors by bacterial surface proteases. IM, inner membrane; LF, lactoferrin; LPS, lipopolyssacharide; OM, outer membrane; T3SS, type 3 secretion system. Created with Biorender.com.

LF can not only directly degrade bacterial virulence factors, it could also induce degradation of virulence factors by surface proteases, present on the bacterial outer membrane (Figure 3) (34). The mechanism behind this is not yet fully elucidated, but it has been suggested that the disruption of the outer membrane by LF exposes virulence factors to surface proteases (34). Which bacterial proteases are then involved in the actual degradation of the bacterial virulence factors, however, is unclear. Lastly, LF has been shown to bind to some virulence factors, such as cable pili of *B. cenocepacia* and several studies report that LF decreases the adherence or invasion of pathogens in host cells (30, 33, 89, 91–96). Hence, it would be interesting to see whether LF interacts with or degrades the involved virulence factors.

The insights obtained from future research on the proteolytic activity of LF, proposed above, could inform the development of practical applications, such as new antibacterial therapies and strategies to combat bacterial infections. The proteolytic activity of lactoferrin could be harnessed, either directly or through the engineering of LFs with enhanced proteolytic activity, to specifically target key bacterial virulence factors in certain infectious diseases. This approach aims to reduce bacterial adherence or invasion into host cells, thereby lowering bacterial pathogenicity. This could be particularly useful not only to treat infectious diseases but also to prevent bacterial colonisation of wounds, medical devices, and prosthetics. Moreover, LF’s proteolytic activity could also be used to treat antibiotic-resistant strains or to complement existing antimicrobial treatments, lowering the chance of developing antimicrobial resistance. In addition, investigating whether lactoferrin can degrade similar proteins across bacterial species, could lead to the creation of broad-spectrum bacterial agents. This could be particularly useful when treating polymicrobial infections or infections where the causative pathogen is unknown. Overall, these potential applications underscore the importance of further research into the proteolytic activity of lactoferrin and its relevance to combat bacterial infections.

In conclusion, further exploration of the proteolytic activity of LF towards bacterial virulence factors will provide us with valuable information to understand its mechanism and its potential targets. Moreover, future research could also reveal whether we can use LF’s proteolytic activity to reduce the pathogenicity of bacterial organisms, leading to the potential development of practical applications to combat bacterial diseases.

## Author contributions

RO: Writing – original draft, Writing – review & editing, Conceptualization. MD: Funding acquisition, Writing – original draft, Writing – review & editing. DV: Funding acquisition, Writing – original draft, Writing – review & editing. EC: Conceptualization, Funding acquisition, Writing – original draft, Writing – review & editing. BD: Conceptualization, Funding acquisition, Writing – original draft, Writing – review & editing.
